# Multi-omics reveals the role of ENO1 in bladder cancer and constructs an epithelial-related prognostic model to predict prognosis and efficacy

**DOI:** 10.1038/s41598-024-52573-8

**Published:** 2024-01-25

**Authors:** Zhixiong Su, Lijie You, Yufang He, Jingbo Chen, Guifeng Zhang, Zhenhua Liu

**Affiliations:** Department of Oncology, Shengli Clinical Medical College of Fujian Medical University, Fujian Provincial Hospital, No. 134, East Street, Fuzhou, 350001 Fujian People’s Republic of China

**Keywords:** Bladder cancer, Tumour biomarkers, Tumour heterogeneity, Tumour immunology, Urological cancer

## Abstract

α-Enolase (*ENO1*) is a crucial molecular target for tumor therapy and has emerged as a research hotspot in recent decades. Here, we aimed to explore the role of *ENO1* in bladder cancer (BLCA) and then construct a signature to predict the prognosis and treatment response of BLCA. Firstly, we found *ENO1* was highly expressed in BLCA tissues, as verified by IHC, and was associated with poor prognosis. The analysis of the tumor immune microenvironment by bulk RNA-seq and scRNA-seq showed that *ENO1* was associated with CD8+ T-cell exhaustion. Additionally, the results in vitro showed that *ENO1* could promote the proliferation and invasion of BLCA cells. Then, the analysis of epithelial cells (ECs) revealed that *ENO1* might promote BLCA progression by metabolism, the cell cycle and some carcinogenic pathways. A total of 249 hub genes were obtained from differentially expressed genes between *ENO1*-related ECs, and we used LASSO analysis to construct a novel signature that not only accurately predicted the prognosis of BLCA patients but also predicted the response to treatment for BLCA. Finally, we constructed a nomogram to better guide clinical application. In conclusion, through multi-omics analysis, we found that *ENO1* was overexpressed in bladder cancer and associated with poor prognosis, CD8+ T-cell exhaustion and epithelial heterogeneity. Moreover, the prognosis and treatment of patients can be well predicted by constructing an epithelial-related prognostic signature.

## Introduction

Bladder cancer (BLCA) is one of the most common malignant tumors in the genitourinary system, with approximately 430,000 cases per year worldwide^1,2^. Its incidence is four times higher in men than in women, and it ranks as the sixth most frequently diagnosed malignancy among men^3^. Contributing to the progress in early detection, the majority of cases are resectable at diagnosis, with a 5-year survival rate of up to 77.1%, whereas approximately 50–70% of those will suffer recurrence within 5 years^4^. In addition, 10–15% of patients encounter metastasis at the time of diagnosis, with a 5-year survival rate as low as 4.75^2^. Tumor heterogeneity, regardless of whether it is at either the molecular or cellular level, results in this apparent divergence^5^. Therefore, it is imperative to search for new therapeutic targets or biomarkers in the management of BLCA.

α-enolase (*ENO1*), also known as 2-phospho-d-glycerate hydrolase, is a glycolytic enzyme that catalyzes the conversion of 2-phosphoglycerate to phosphoenolpyruvate during glycolysis^6^. Studies have confirmed that *ENO1* is overexpressed in more than 70% of cancers and is also associated with poor patient outcomes^7–9^. It can promote cancer progression by sustaining proliferative signaling^10,11^, activating invasion and metastasis^10,12,13^, inducing angiogenesis^14^, deregulating cellular energy^15^ and avoiding immune destruction^16,17^. Additionally, ENO1 was reported to reduce tumor cell tolerance to hypoxia via the aerobic glycolysis pathway and resulted in tumor angiogenesis^18,19^. However, previous studies have mostly focused on cell lines, making it difficult to reveal the true tumor microenvironment. With the development of technology, single-cell RNA sequencing (scRNA-seq) is used to define the global gene expression profile of a single cell, which allows us to explore the hidden heterogeneity in a cell population^20^. Hence, we sought to reveal the role of *ENO1* in BLCA through scRNA-seq and bulk RNA-seq data.

In the present study, we showed that *ENO1* was overexpressed in BLCA tissues and was correlated with poor prognosis in BLCA patients. Then, we found that *ENO1* may be associated with CD8+ T-cell exhaustion and that there was heterogeneity in epithelial cells (ECs) with different *ENO1* expression levels. Next, we screened the hub genes by differential expression analysis and constructed a novel signature to predict the prognosis and response to treatments in BLCA patients. Finally, we developed a nomogram combining the novel signature and clinicopathological features to facilitate its clinical application in the prognosis of BLCA.

## Materials and methods

### Patients and datasets

The Cancer Genome Atlas (TCGA, https://portal.gdc.cancer.gov/) bladder cancer RNA-seq profiling in the form of fragments per kilobase million (FPKM) and corresponding clinicopathological data were collected from the TCGA database. The RNA-seq data and clinicopathological data of GSE13507 were obtained from the Gene Expression Omnibus (GEO) database. We removed patients with a survival time of 0. Finally, 403 patients in TCGA-BLCA and 165 patients in GSE13507 were used in this study. The RNA-seq profiles and clinical data of the “IMvigor 210” cohort were derived from http://research-pub.gene.com/IMvigor210CoreBiologies/^21^. Furthermore, 10 pairs of frozen, surgically resected tumor specimens were acquired from patients pathologically diagnosed with BLCA at the Fujian Provincial Hospital (FPH) between December 2015 and December 2017. This study was approved by the ethics committee of the FPH. The clinical and pathological characteristics of the above patients are shown in Supplementary Table 1.

### scRNA-seq data quality control and processing

We downloaded the scRNA-seq data of CD8+ T cells in GSE149652, which included 10,763 CD8+ T cells, to explore the relationship between *ENO1* and CD8+ T cells^22^. The scRNA-seq profiling of 7 primary BLCA cases with no treatment was obtained from GSE135337 to analyze the heterogeneity of malignant epithelial cells (ECs)^23^. Using the “Seurat” package^24^, we excluded cells with less than 200 genes detected and more than 10% mitochondrial gene proportion. Based on the top 1500 variable genes across all cell samples, PCA and t-SNE were then performed to classify the cell samples, and the marker genes were screened with |log2FC|> 0.5 and adjusted P < 0.05 filtering^25^. The results of quality control in GSE149652 and GSE135337 are shown in supplementary Fig. 1 and supplementary Fig. 2, respectively. Finally, we obtained 10,483 cells in GSE149652 and 36,695 cells in GSE135337.

Then, we annotated the cell categories in GSE149652 based on the marker genes with CellMarker (http://biocc.hrbmu.edu.cn/CellMarker/) ^26^ and annotated the cell categories in GSE135337 by using the R package “singleR”^27^. In GSE135337, we further screened 35,945 ECs after using “singleR” annotation for follow-up analysis. The R package “Monocle2” was used to perform trajectory analysis^28^. The R package “scMetabolism” was used to calculate the activity score of metabolic pathways^29^. In addition, the intersection of differential genes between different ECs were taken as hub genes.

### Differential expression and prognosis analysis of *ENO1*

Using the “limma” and “survminer” R packages, we analyzed the differential expression between normal and tumor tissues and the prognostic value of *ENO1*. Furthermore, we used immunohistochemistry (IHC) to verify the difference in ENO1 protein expression between tumor and normal tissues. IHC staining of paraffin-embedded tissues with an antibody against *ENO1* (1:200, 11, 204-1-AP, Proteintech) was performed according to the standard procedures as previously described and was evaluated by two independent pathologists. As described previously, the percentage (P) and intensity (I) of cytoplasmic or membrane expression were multiplied to generate the H score (H score = P*I)^30^.

### Cell culture and transfection

The human BLCA cell line HT1376 was cultured in RPMI-1640 medium (Invitrogen) at 37 °C with 5% CO_2_. All media were supplemented with 10% FBS. For the knockdown assay, small interfering RNAs targeting ENO1 (si-ENO1-296, si-ENO1-664, si-ENO1-791 and si-ENO1-880) were applied, and scramble siRNAs (siNC) were used as the negative control. The siRNA sequences targeting ENO1 can be found in Supplementary Table 2.

### Western blot assays

Antibodies against ENO1 (1:1000, XC1033) were purchased from BIOTECH. Briefly, cells were lysed by RIPA buffer with protease and phosphatase inhibitor cocktail following the manufacturer’s specification, and then the concentrations were measured and normalized by BCA assay. Western blotting was performed according to the standard methods as depicted in the manufacturer’s specification and previous studies^31^.

### Quantitative real-time PCR

Total RNA was isolated from the cultured cells using TRIzol reagent (TaKaRa). RNA was then converted into cDNA by applying the TaKaRa PrimeScript™ RT Master Mix (Perfect Real Time) Kit. A ChamQ Universal SYBR qPCR Master Mix Kit from Norvezan was used to examine gene mRNA expression levels using a PCR instrument (Thermo Fisher). The specific primer sequences are listed in Supplementary Table 2.

### Cell counting kit-8 assay

Cells were plated in 96-well plates at 1*10^4^ per well, and 10 µl of CCK-8 solution (A311, Vazyme) was added to each well after 24 and 48 h. Then, the cells were incubated for 1 h at 37 °C and 5% CO_2_. Finally, the optical absorbance was measured at 450 nm. Each experiment was performed in triplicate.

### Wound healing assay

Cells from each group were plated into 6-well plates at approximately 95% confluence. Then, we used a 200 µl pipette tip to make symmetrical wounds. After being washed twice with PBS, the cells were incubated with serum-free RPMI-1640 medium for 24 h (or 48 h). Migration pictures were taken at 0 h and 24 h (or 48 h) after drawing the wound. The wound distance of each group at 40× magnification was measured by ImageJ software. Each experiment was performed in triplicate.

### TME and immunotherapy benefits analysis

The “ESTIMATE” R package was utilized to calculate the immune scores, stromal scores, and ESTIMATE scores, which can be used to evaluate the abundance of immune cells and stromal cells in the tumor microenvironment (TME). The infiltration and function of immune cells were quantified by single-sample gene set enrichment analysis (ssGSEA) via the “gsva” R package^32^. Correlation analysis between the CD8+ T-cell infiltration level and *ENO1* expression or prognosis in bladder cancer patients was obtained from the Tumor Immune Estimation Resource (TIMER, https://cistrome.shinyapps.io/timer/) website^33^. Immune activity scores of anticancer immunity across the seven-step cancer-immunity cycle for BLCA patients were downloaded from the website Tracking Tumor Immunophenotype (TIP, http://biocc.hrbmu.edu.cn/TIP/index.jsp) ^34^. Immunophenoscore (IPS) scores were obtained from the Cancer Immuneome Database (TCIA, https://tcia.at/home), which can predict patient response to immunotherapy^35^.

### Functional analysis

Gene set enrichment analysis (GSEA), Gene Ontology biological processes (GO-BP) and Kyoto Encyclopedia of Genes and Genomes (KEGG) analysis^36–38^ were used to analyze the main functions using the R package “clusterProfiler”^39^.

### Consensus clustering analysis

We explored the prognostic value of hub genes by clustering TCGA-BLCA patients into different clusters using the “ConsensusClusterPlus” R package^40^. The cumulative distribution function (CDF) and delta area were considered to determine the optimal number of groups (k). A relatively high consistency, a low variation coefficient and a significant increase in the CDF curve area were used to determine the cluster number. Then, we used GSE13507 to verify the results of the consensus clustering analysis.

### Construction and validation of a prognostic signature in BLCA

We screened the prognostically significant hub genes (P < 0.05) by univariate Cox regression analysis. Least absolute shrinkage and selection operator (LASSO) regression is known to be able to remove unimportant variables via the regression coefficients penalizing the size of the parameters. Applying the LASSO regression method, feature selection and predictive signature building was done^41^. Therefore, we constructed a novel signature via LASSO analysis to predict the overall survival (OS) of BLCA patients in the TCGA cohort using the R package “glmnet”^42^. The risk score for each patient was determined using the following formula:

$${\text{Risk score}} = \mathop \sum \limits_{i = 0}^{n} Coef\left( i \right) \times x\left( i \right)$$

Thereafter, the patients were classified into low- and high-risk groups based on the median risk score. We determined the prognostic ability of the novel signature by generating Kaplan–Meier survival curves and receiver operating characteristic (ROC) curves using the R packages “survminer” and “survivalROC”. The GSE13507 cohort was used to verify the prognostic performance of the novel signature in the same manner as mentioned above.

### Drug sensitivity analysis

The sensitivity of BLCA patients to each commonly used drug was calculated by IC50 values using the “pRRophetic” package, and the corresponding data were obtained from the GDSC database^43^.

### Construction of the nomogram

Univariate and multivariate Cox regression were used to screen the independent prognostic factors for BLCA patients in TCGA, based on which we constructed a nomogram using the “rms” package. Additionally, we evaluated this nomogram using calibration curves, ROC curves, and decision-making curves (DCAs).

### Statistical analysis

The normality of the variables was tested by the Shapiro‒Wilk test. Differences between two normally distributed groups were determined by Student’s t test, and the Wilcoxon test measured differences between two nonnormally distributed variables. One-way analysis of variance (ANOVA) tests were used as a parametric method for multiple group comparisons, while Kruskal–Wallis tests were used as a nonparametric method. Based on correlation coefficients, Pearson correlation and distance correlation analyses were performed. Chi-square contingency tests were used for contingency table analyses. The Benjamini‒Hochberg method was applied for P values to FDR conversion in the DEG analysis. All tests were two-sided, and P < 0.05 was considered statistically significant. All statistical analyses were performed using RStudio version 4.1.0, and a two-sided P < 0.05 was deemed statistically significant.

## Result

### *ENO1* was overexpressed in BLCA and associated with poor prognosis

**Figure 1:**
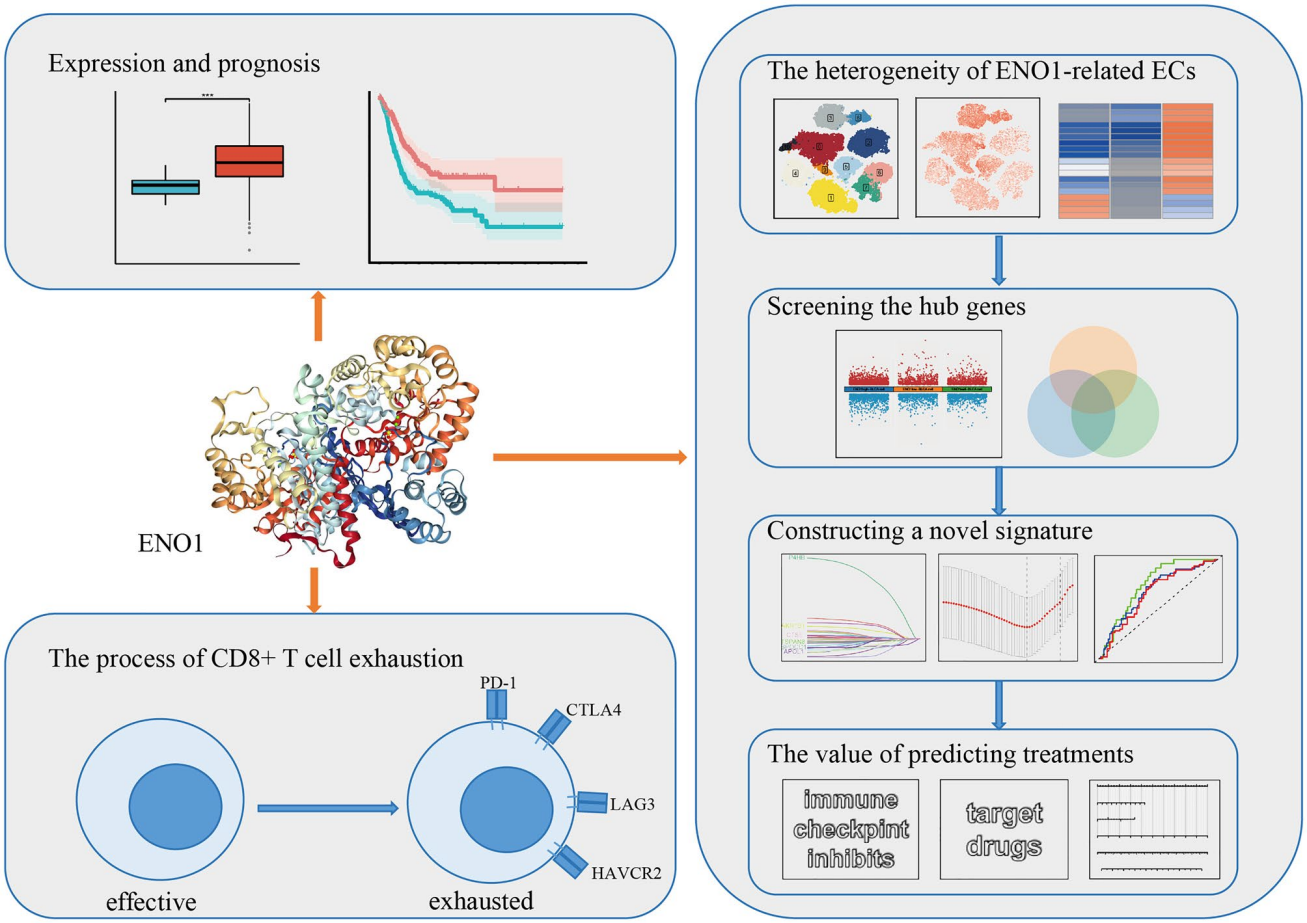
Flow chart of this study.

**Figure 2:**
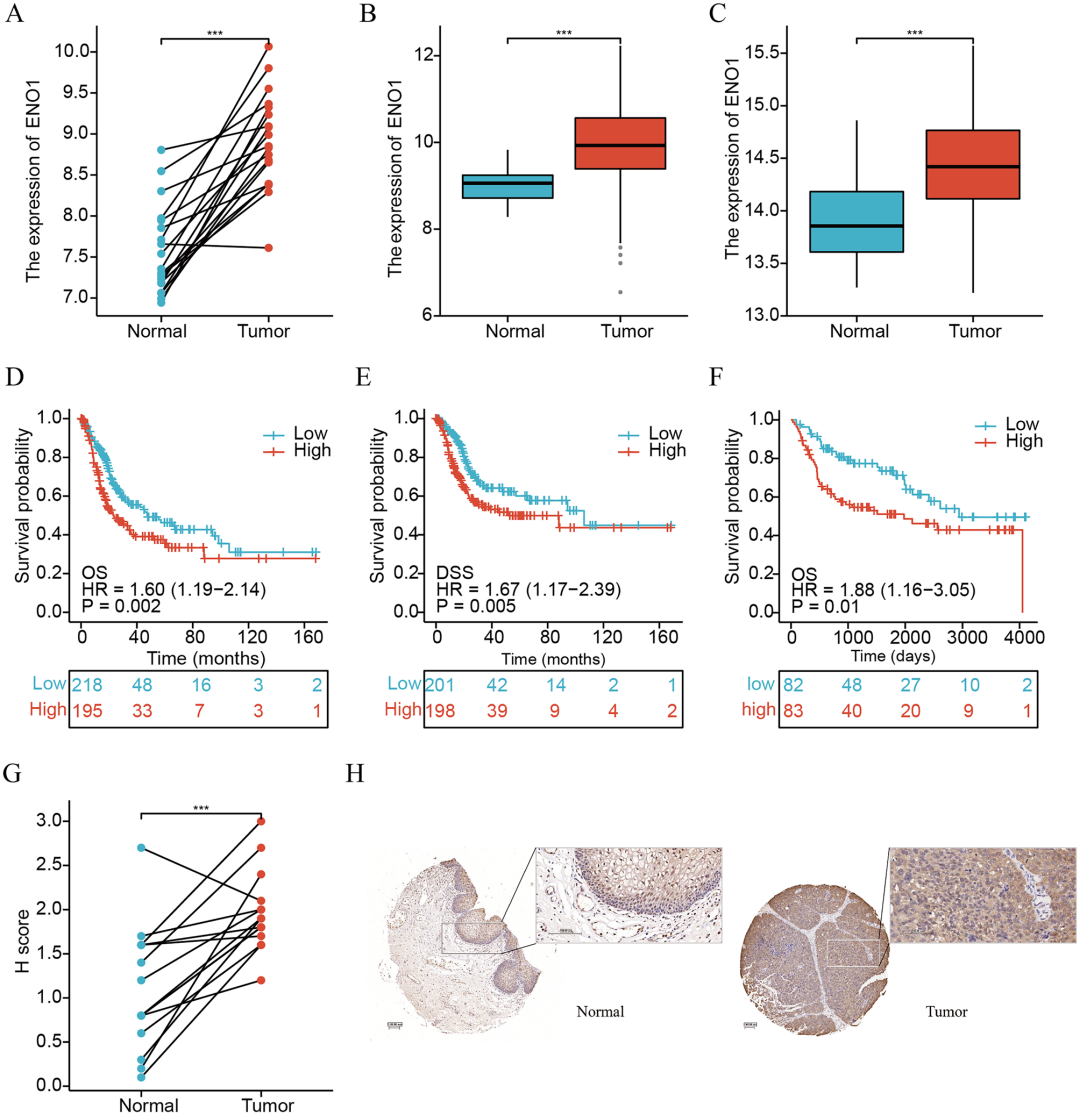
*ENO1* was overexpressed in BLCA tissues and associated with poor prognosis. Differential expression among paired samples (**A**) and unpaired samples (**B**) in the TCGA cohort. (**C**) Differential expression among unpaired samples in the GSE13507 cohort. Kaplan–Meier survival curve of OS (**D**) and DSS (**E**) between the high- and low-expression groups of *ENO1* in the TCGA cohort. (**F**) Kaplan‒Meier survival curve of OS between the high- and low-expression groups of *ENO1* in the GSE13507 cohort. (**G**) Differential H score among paired samples by IHC in the PFH cohort. (**H**) Typical staining histochemical results of normal and tumor tissues in the PFH cohort. ***P < 0.001; **0.001 < P < 0.01; *0.01 < P < 0.05; ns: P > 0.05.

Figure 1 illustrates the research process of this study. By differential analysis, we observed higher expression of *ENO1* in BLCA tissues than in normal tissues, both in paired and unpaired samples (all P < 0.05; Fig. 2A–C). Based on the median expression of *ENO1*, we classified BLCA patients into low- and high-expression groups. As shown in Fig. 2D–F, the high-expression group was positively correlated with worse OS and disease-specific survival (DSS) in BLCA patients (all P < 0.05). Moreover, to verify the differential expression of *ENO1* protein, we conducted IHC on tumor and adjacent tumor samples of FPH, and the results showed a higher H score for *ENO1* in tumor tissues than in normal tissues (P < 0.05; Fig. 2G). Figure 2H displayed typical results of IHC staining. Collectively, these results suggested that *ENO1* played an essential role in the prognosis of BLCA.

### *ENO1* was relevant to CD8+ T-cell exhaustion

**Figure 3:**
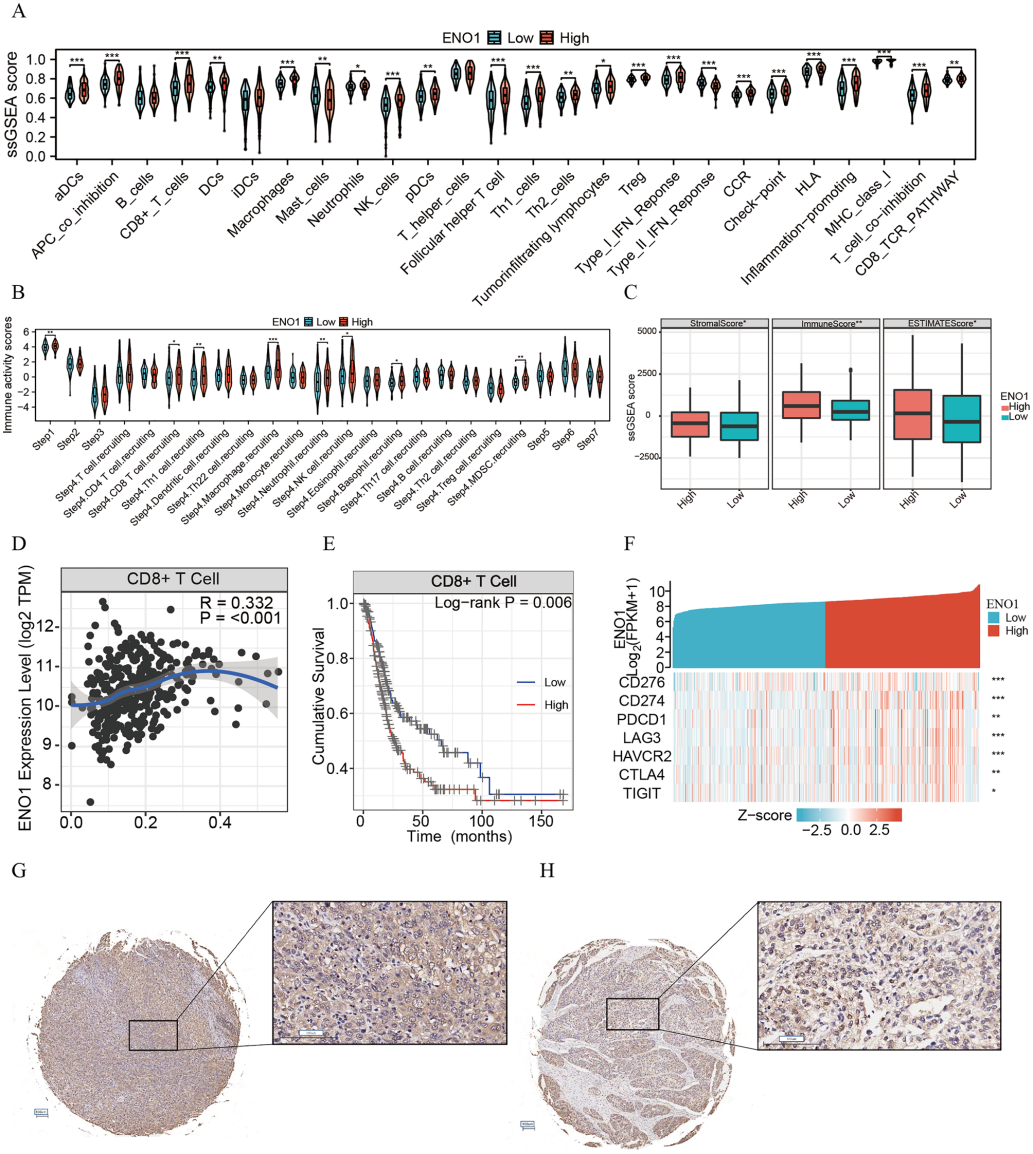
*ENO1* was correlated with a suppressive antitumor microenvironment and exhausted CD8+ T cells. (**A**) The infiltration levels of 16 immune cells and the activation levels of immune-related pathways of the high- and low-expression groups by ssGSEA. (**B**) Differences in cancer immune cycling activity between the high- and low-expression groups. (**C**) Differences in ESTIMATE score, immune score and stromal score among the high- and low-expression groups. (**D**) Scatter plot of the association between *ENO1* and CD8+ T cells. (**E**) Kaplan‒Meier survival curve of OS between the high- and low-infiltrating level groups of CD8+ T cells. (**F**) Heatmap showing the relationship between *ENO1* and T-cell exhaustion markers. Typical staining histochemical results of *PD-1* expression greater than 50% (**G**) and *PD-1* expression less than 1% (**H**) in the PFH cohort. ***P < 0.001; **0.001 < P < 0.01; *0.01 < P < 0.05; ns: P > 0.05.

To investigate the influence of *ENO1* on the TME, we explored the correlation between *ENO1* expression and immune cell infiltration. By ssGSEA, we found that most of the 16 infiltrating immune cells, including CD8+ T cells and Tregs, had a higher abundance of infiltrating immune cells in the high-expression group (all P < 0.05; Fig. 3A). To explore the underlying mechanism, we performed the corresponding immune function analysis, and the results revealed that heightened levels of the CD8 TCR pathway were elevated in the high-expression group, as well as the immunosuppressive pathways of T-cell coinhibition and APC coinhibition (all P < 0.05; Fig. 3A). Furthermore, analysis of immune activity scores across the seven steps of the cancer-immunity cycle showed that the activity score of step 1 and several steps of step 4 was high in the high-expression group (Fig. 3B). However, the antitumor effect phase (steps 5–7) of immune cells was not significantly different (Fig. 3B). In addition, we found that samples in the high-expression groups had significantly higher immune scores and ESTIMATE scores than those in the low-expression group (all P < 0.05; Fig. 3C). These findings suggested that the activity of antitumor immune cells may be suppressed in high-expression group patients.

To further explore the mechanism, we found that CD8+ T cells were positively correlated with *ENO1* expression (R = 0.332, P < 0.001; Fig. 3D) and associated with poor prognosis (P < 0.05; Fig. 3E) in BLCA using TIMER website analysis. T-cell exhaustion (TEX) is characterized by poor effector functions and increased expression of inhibitory receptors such as *CD274, CD276, PDCD1, CTLA4, TIGIT, HAVCR2* and *LAG3*^44^. Therefore, we explored the relationship between ENO1 and inhibitory receptors, which suggested that *ENO1* was positively correlated with the expression of inhibitory receptors (all P < 0.05; Fig. 3F). Furthermore, we obtained IHC images of *ENO1* protein with different *PD-1* expression levels and found that patients with high *PD-1* expression had higher *ENO1* expression (Fig. 3G, H). Consequently, we hypothesized that the highly infiltrated CD8+ T cells in the high-expression group may have been exhausted.

**Figure 4:**
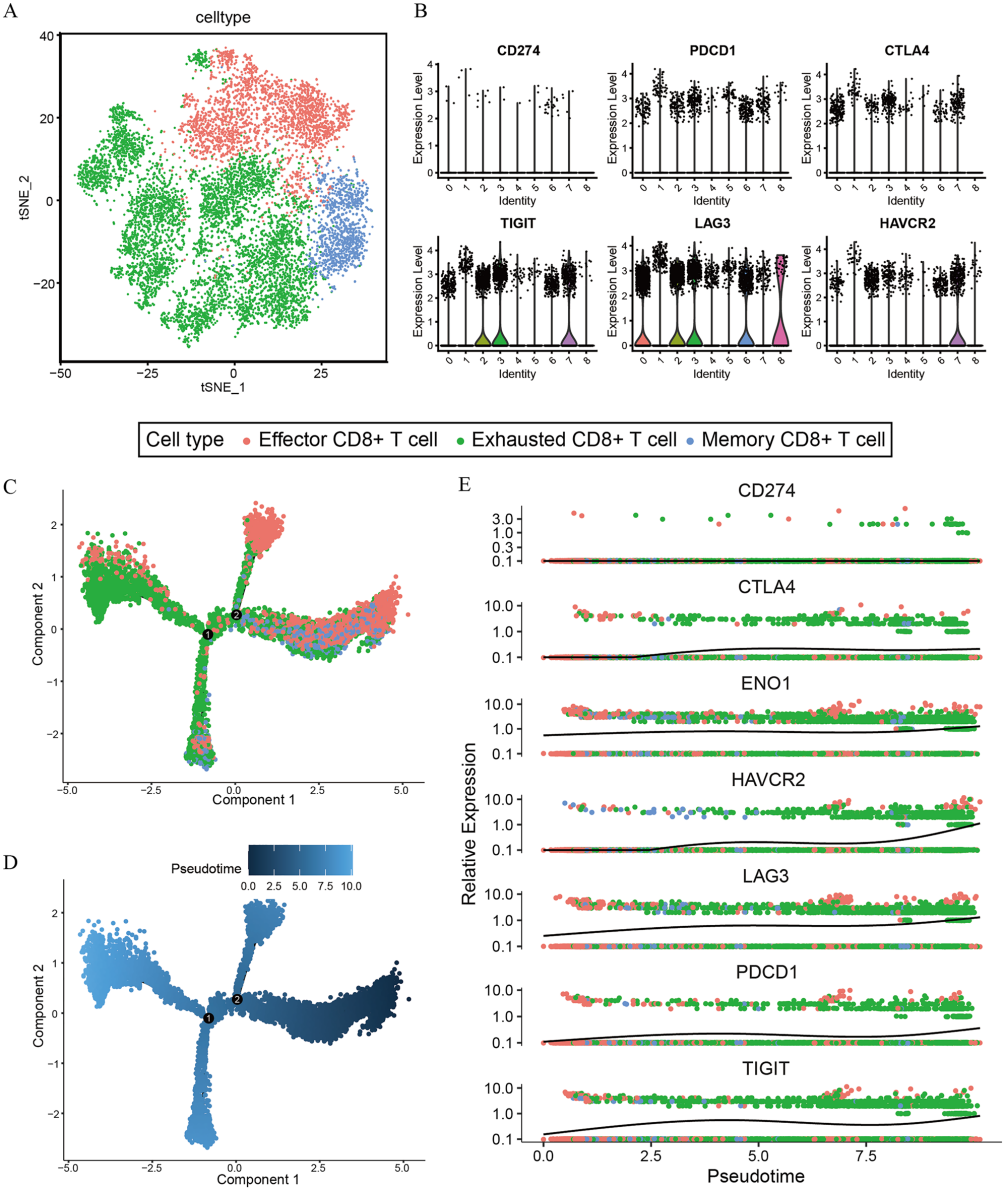
*ENO1* was associated with exhausted CD8+ T cells in BLCA by single-cell analysis. (**A**) Annotations for CD8+ T-cell subtypes. (**B**) Differential expression of immunosuppressive receptors between clusters 0–9. (**C**, **D**) The results of pseudotime analysis in different CD8+ T-cell subtypes. (**E**) The expression of *ENO1* and immunosuppressive receptors increased with pseudotime.

Given that CD8+ T cells may have been exhausted in the *ENO1* high-expression group, we tried to explore the association between *ENO1* and exhausted CD8+ T cells in the BLCA scRNA-seq dataset. The t-SNE map showed that CD8+ T cells were divided into 9 different clusters (Fig. 4A). By annotating the marker genes of different t-SNE clusters, we found that clusters 0, 2, 3, 6, 7 and 8 were “exhausted CD8+ T cells” (including 6564 cells), clusters 1 and 5 were “effector CD8+ T cells” (including 2752 cells), and cluster 4 was “memory CD8+ T cells” (including 1167 cells) (Supplementary Fig. 1E, F). Then, we analyzed the expression of immunosuppressive receptors (*CD274, PDCD1, CTLA4, TIGIT, LAG3* and *HAVCR2*) between different clusters and found that these genes were more highly expressed in exhausted CD8+ T cells than in effector CD8+ T cells and memory CD8+ T cells (Fig. 4B). We next established a pseudotemporal trajectory to trace cell differentiation. The CD8+ T-cell trajectory was reconstructed using Monocle2, which revealed that the pseudotime increased from effector CD8+ T cells and memory CD8+ T cells to exhausted CD8+ T cells (Fig. 4C, D). Moreover, we explored the trend of *ENO1* and immunosuppressive gene expression over pseudotime and found that their expression increased gradually (Fig. 4E). Hence, we believed that the increase in *ENO1* was associated with the process of CD8+ T-cell exhaustion.

### *ENO1* knockdown inhibits cell proliferation and invasion in BLCA

**Figure 5:**
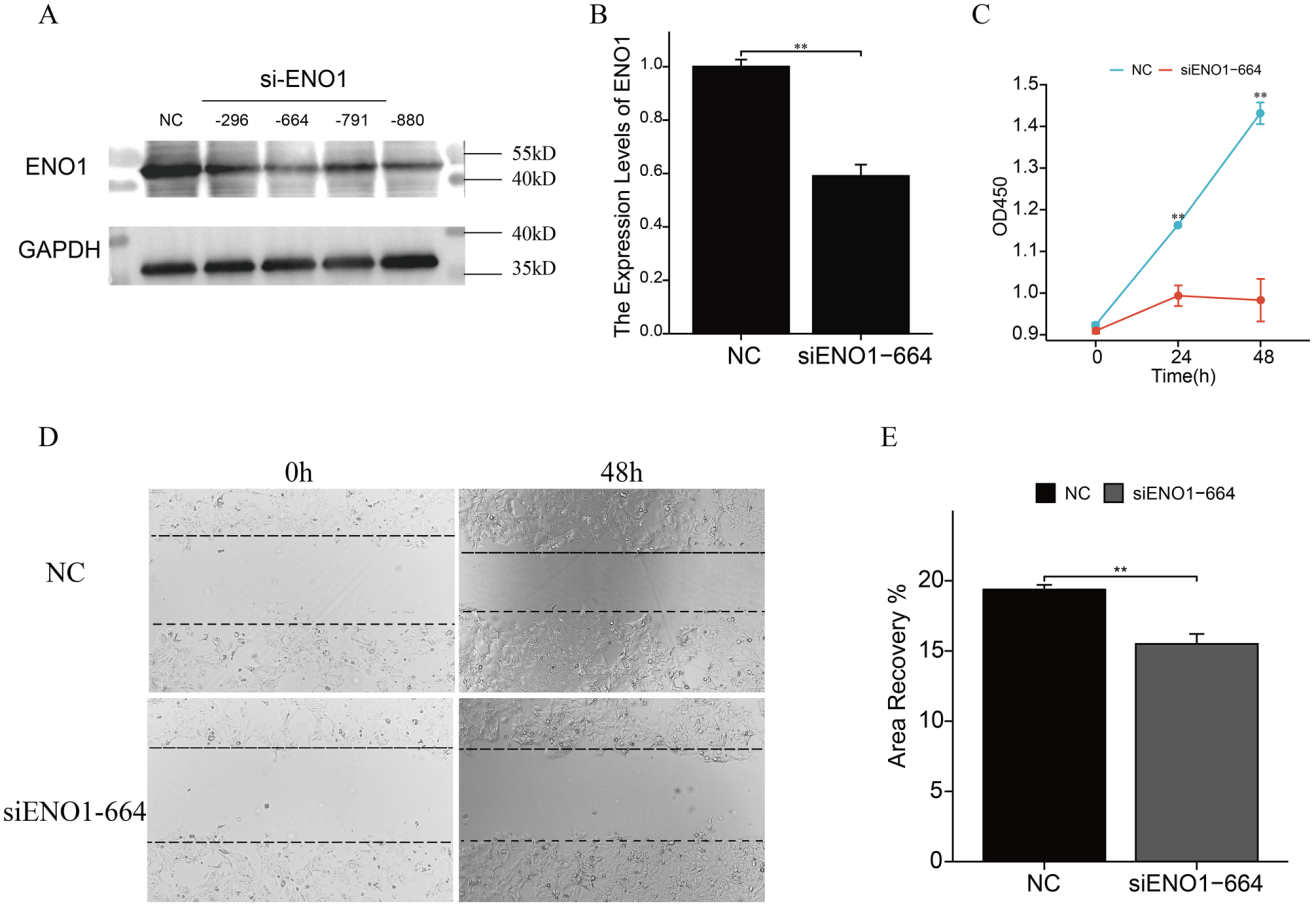
*ENO1* knockdown inhibits cell proliferation and invasion. (**A**, **B**) shENO1-664 significantly inhibited the expression of *ENO1* in HT1376 cells (original blots are presented in Supplementary Fig. 5). (**C**) The results of CCK-8 assays. (**D**, **E**) The results of wound healing assays. ***P < 0.001; **0.001 < P < 0.01; *0.01 < P < 0.05; ns: P > 0.05.

To explore the role of *ENO1* in BLCA cells, we performed CCK-8 and wound healing assays in HT1376 cell lines in vitro. First, we transfected different siRNAs into HT1376 cell lines and verified *ENO1* knockdown by western blotting (Fig. 5A) and qRT‒PCR (P < 0.05; Fig. 5B). Next, we selected siENO1-664 for further analysis. The CCK-8 assay showed that knockdown of *ENO1* significantly inhibited the proliferation of HT1376 cells (P < 0.05; Fig. 5C). In addition, the wound healing results suggested that *ENO1* knockdown inhibited the invasion ability of cells (P < 0.05; Fig. 5D, E). In conclusion, these in vitro results confirmed that the proliferation and invasion abilities of BLCA cells were correspondingly impaired after *ENO1* expression was decreased.

### *ENO1*-related EC subgroups have heterogeneity

**Figure 6:**
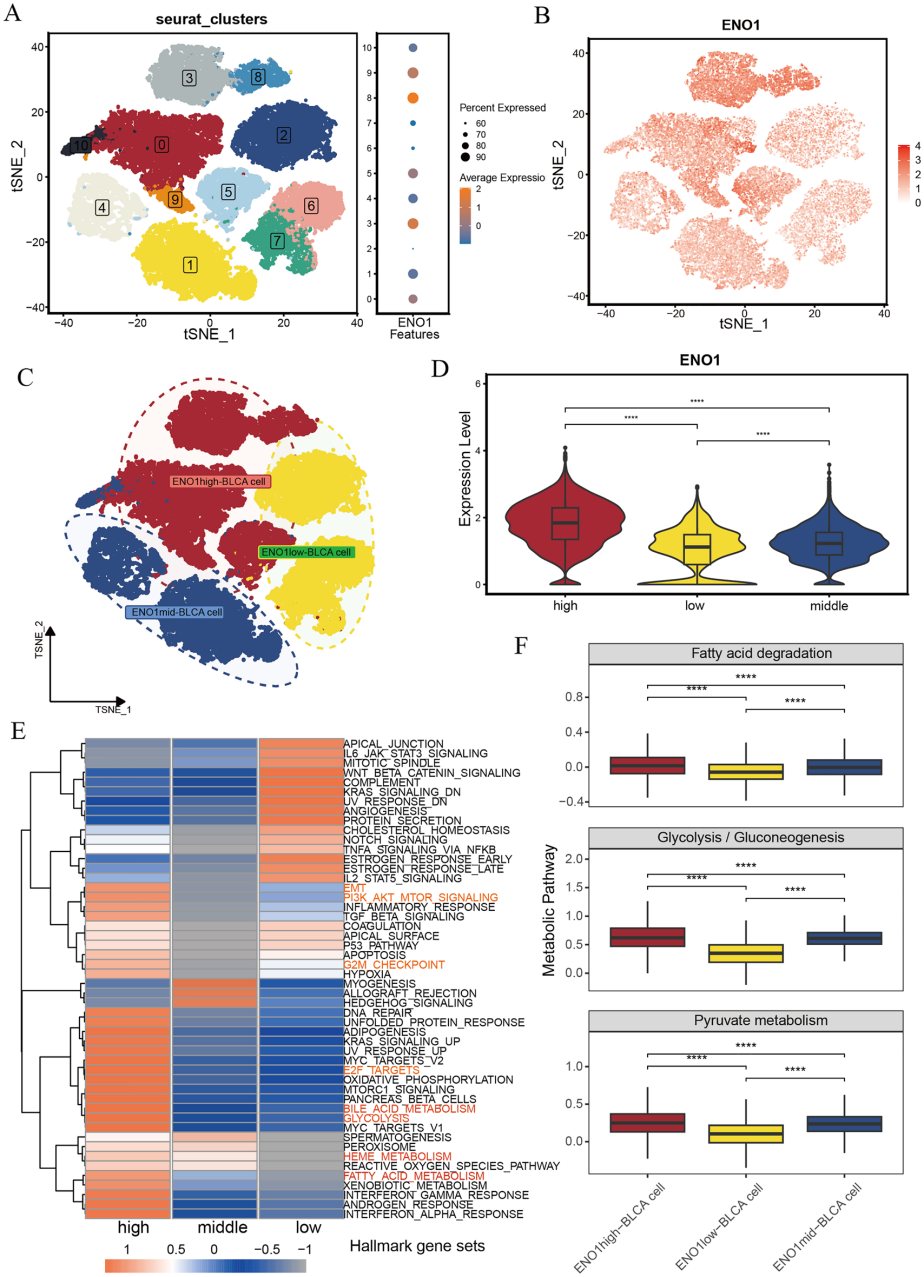
There was heterogeneity between ECs with different ENO1 expression levels. (**A**) Epithelial Cells were classified into 11 clusters with the t-SNE algorithm in GSE135337. (**B**) The tSNE map shows the expression levels in every cell. (**C**) Annotations for epithelial cell subtypes. (**D**) The differential expression of ENO1 in different subtypes of epithelial cells. (**E**) The heatmap shows the activities of hallmark pathways. (**F**) The differential activity of several metabolic pathways between different subtypes of epithelial cells. ***P < 0.001; **0.001 < P < 0.01; *0.01 < P < 0.05; ns: P > 0.05.

Considering that *ENO1* was highly expressed in tumor cells and positively correlated with malignant phenotypes, we performed further analysis of epithelial cells (ECs) in the scRNA-seq data. First, we annotated 35945 ECs from GSE135337 (Supplementary Fig. 2). Then, we reclassified ECs into 11 clusters through t-SNE analysis and found that clusters 0, 3, 5, 8, and 9 had high *ENO1* expression, clusters 1, 4, and 10 had moderate *ENO1* expression, and clusters 2, 6, and 7 had low *ENO1* expression (Fig. 6A). The t-SNE map showed the expression of *ENO1* in every ECs (Fig. 6B). Based on the expression of *ENO1*, we annotated ECs as *ENO1*^high^-BLCA cells, *ENO1*^mid^-BLCA cells and *ENO1*^low^-BLCA cells (Fig. 6C), and Fig. 6D shows the differential expression of *ENO1* between them (all P < 0.05). To explore potential heterogeneity between different ECs, we performed GSVA. The results showed that* ENO1*^high^-BLCA cells had higher energy metabolism pathway activity (including glycolysis, fatty acid metabolism, bile acid metabolism and heme metabolism), higher cell cycle-related pathway activity (including the G2M checkpoint and E2F targets) and higher epithelial-mesenchymal transition (EMT) and PI3K/AKT/mTOR pathway activity (Fig. 6E). In addition, through metabolic activity analysis, we found that *ENO1* expression was positively correlated with the activities of fatty acid degradation, glycolysis and pyruvate metabolism pathways (all P < 0.05, Fig. 6F). These results suggested that there was heterogeneity among ECs with different *ENO1* expression levels and that *ENO1* may contribute to poor prognosis by enhancing EC energy metabolism, regulating the cell cycle, and promoting EMT.

### Obtaining the hub genes between different ECs

**Figure 7:**
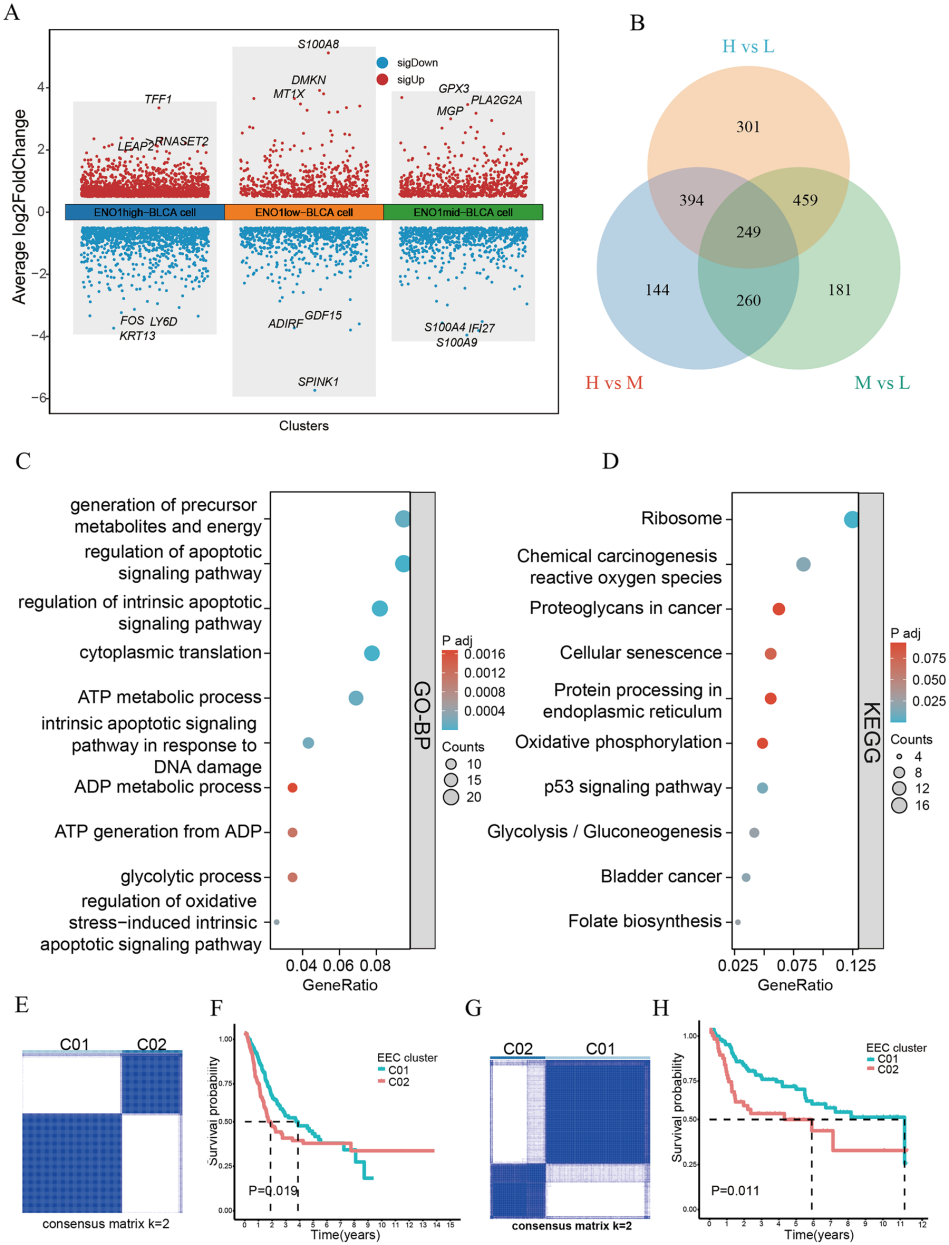
The function of hub genes and prognostic value. (**A**) The Manhattan map shows the differentially expressed genes. (**B**) Venn diagram showing the intersection between different differentially expressed genes. (**C**) The results of GO-BP analysis. (**D**) The results of KEGG analysis. (**E**) The consensus heatmap shows that the BLCA patients were divided into two distinct clusters when k = 2 in TCGA. (**F**) Kaplan‒Meier survival curve of OS between different clusters in TCGA. (**G**) The consensus heatmap shows that the BLCA patients were divided into two distinct clusters when k = 2 in GSE13507. (**H**) Kaplan‒Meier survival curve of OS between different clusters in GSE13507.

The Manhattan map showed that genes were upregulated and downregulated between different ECs (Fig. 7A). By taking the intersection, we obtained 249 hub genes (Fig. 7B). GO-BP analysis showed that hub genes were mainly concentrated in energy metabolism and apoptosis regulation (Fig. 7C), and KEGG analysis showed that hub genes were involved in energy metabolism, ribosome function and carcinogenic pathways (Fig. 7D). To probe the effect of prognosis from hub genes, we performed consensus clustering analysis in the TCGA cohort. We observed a relative change in the CDF of the consensus cluster from k = 2 to k = 9 (Supplementary Fig. 3A); the delta area under the CDF curve from k = 2 to 9 is depicted in Supplementary Fig. 3A. The corresponding heatmap presents the results of this consensus from k = 2 to 9 (k = 2, Fig. 7E; k = 1, k = 3–9, Supplementary Fig. 3A). The optimal cluster number was thus determined to be k = 2 after comprehensive consideration. Then, a significant difference in OS was observed between patients C01 and C02 (P = 0.019; Fig. 7F). In addition, we conducted the above analysis on GSE13507 and found that the results were consistent with those of the TCGA cohort (Fig. 7G, H, Supplementary Fig. 3B).

### Construction and validation of the novel prognostic signature

**Figure 8:**
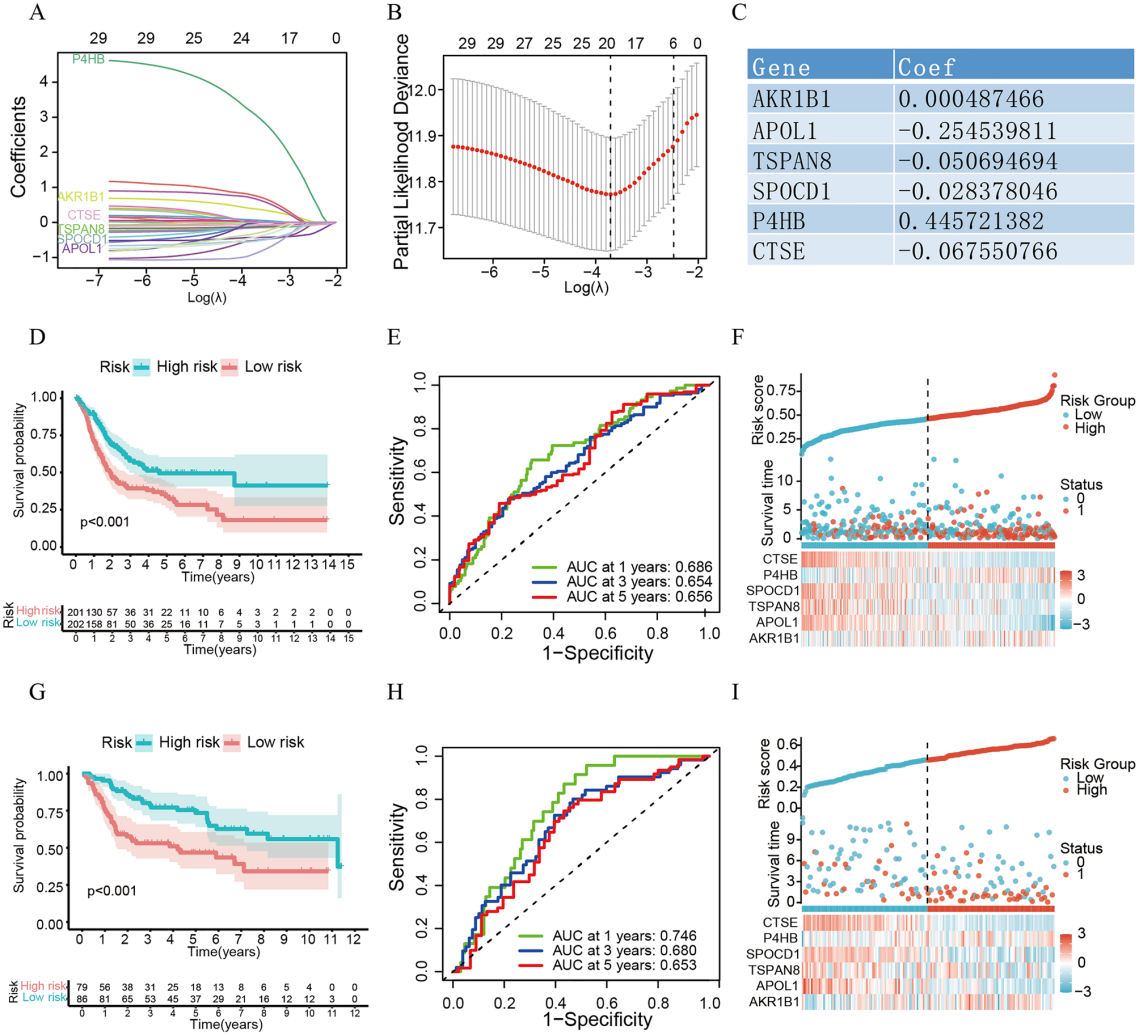
The novel signature can precisely predict the prognosis of BLCA patients. (**A**) LASSO coefficient profiles of the 29 hub genes. (**B**) Plots of the tenfold cross-validation error rates. (**C**) The coefficients of 6 candidate genes. (**D**) Kaplan‒Meier survival curve of OS between the high- and low-risk groups in TCGA. (**E**) The ROC curve of the novel signature in TCGA. (**F**) Distribution of risk score, survival status and genes expressed in TCGA. (**G**) Kaplan‒Meier survival curve of OS between the high- and low-risk groups in TCGA. (**H**) The ROC curve of the novel signature in TCGA. (**I**) Distribution of risk score, survival status and genes expressed in TCGA.

The TCGA cohort was used as the training cohort to evaluate the prognostic value of the above 249 hub genes (Supplementary Table 3), among which 29 hub genes were identified as prognostic genes (all P < 0.05; Supplementary Table 4). Next, among the 29 hub genes, six (*AKR1B1, APOL1, TSPAN8, SPOCD1, P4HB* and *CTSE*) were screened as candidate genes to construct the prognostic prediction signature using LASSO regression analysis (Fig. 8A–C). The risk score of the novel signature was calculated according to the following formula: Risk score = [*AKR1B1* expression*(0.000487466)] + [*APOL1* expression*(− 0.254539811)] + [*TSPAN8* expression*(− 0.050694694)] + [*SPOCD1* expression*(− 0.028378046)] + [*P4HB* expression*(0.445721382)] + [*CTSE* expression*(− 0.067550766)].

Taking the median risk score as the cutoff value, patients in the TCGA set were divided into two subgroups: low- and high-risk. The K‒M survival curve showed that the median OS was significantly shorter in the high-risk subgroup than in the low-risk subgroup (P < 0.001; Fig. 8D). ROC analysis revealed that the novel signature exhibited an excellent predictive capability in the TCGA set (AUC values at 1 year, 3 years and 5 years: 0.686, 0.654, and 0.656, respectively; Fig. 8E). Figure 8F shows the changes in patient survival status and the expression of signature genes with the change in risk score, indicating the accuracy of the signature in predicting prognosis.

The GSE13507 dataset was used as the test cohort to verify the prognostic value of the novel signature, and it was also divided into two different subgroups using the same cutoff value as in the TCGA dataset. A survival discrepancy between the two subgroups was well exhibited using K‒M curves (P < 0.001; Fig. 8G). ROC analysis also suggested that the novel signature could accurately predict the GSE13507 dataset (AUC values at 1 year, 3 years, and 5 years: 0.746, 0.680, and 0.653, respectively; Fig. 8H). Figure 8I also shows the good predictive power of the signature.

### The novel signature can predict the response rates to immunotherapy

**Figure 9:**
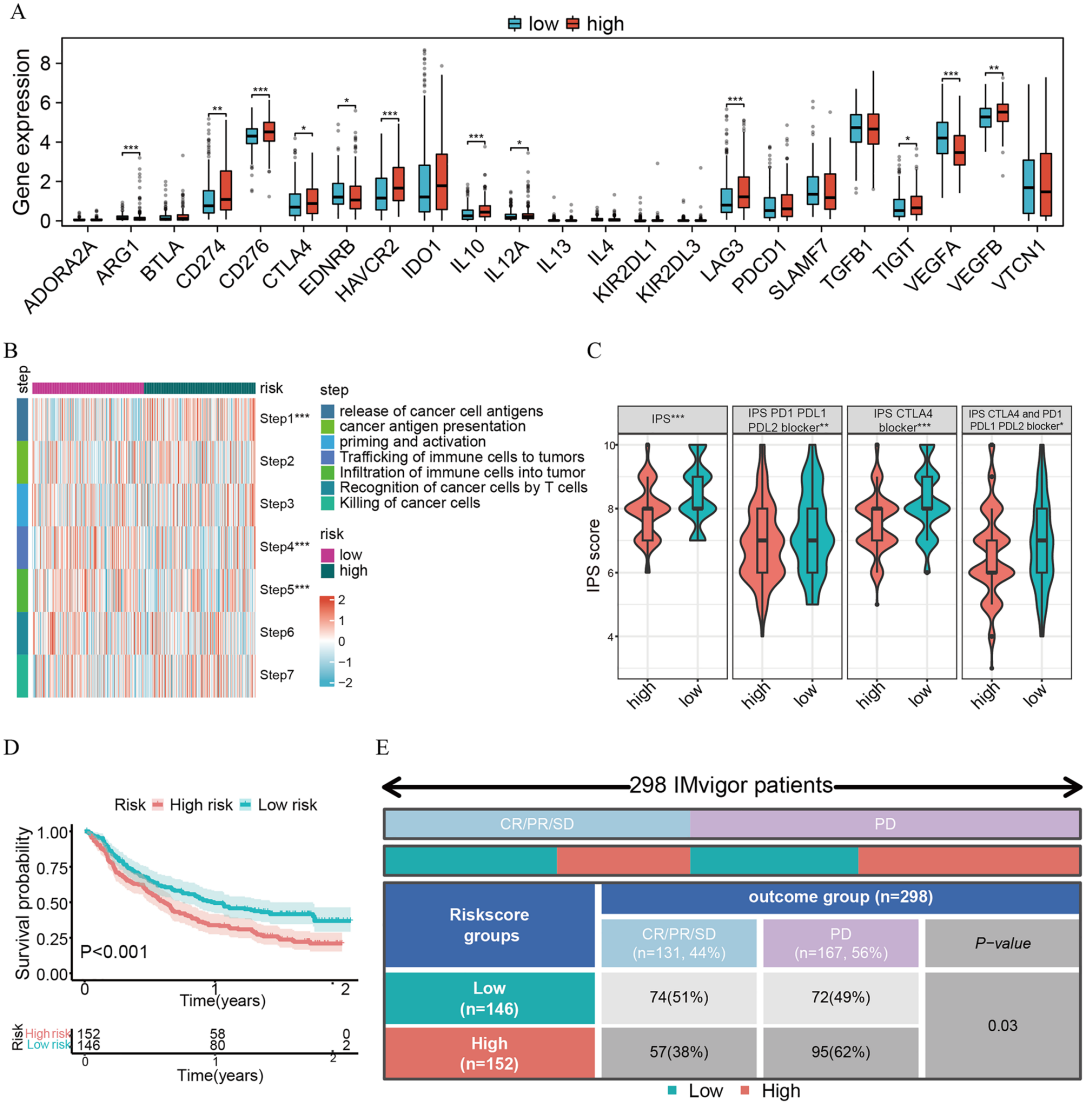
The novel signature can predict the response rates to immunotherapy. (**A**) The differential expression of immune checkpoint genes between high- and low-risk subgroups in TCGA. (**B**) The heatmap shows the differences in the seven steps of the cancer-immunity cycle analysis in TCGA. (**C**) The different IPS scores between high- and low-risk subgroups in TCGA. (**D**) Kaplan‒Meier survival curve of OS between the high- and low-risk groups in the IMvigor 210 cohort. (**E**) Distribution of the immune response to immunotherapy therapy in different risk subgroups in the IMvigor 210 cohort.

To further explore the potential value of the novel signature, we performed an analysis of the relationship between it and immunotherapy in BLCA patients. First, we found it has higher expression of immune checkpoint genes such as *CD274*, *CD276*, and *CTLA4* in high-risk group (Fig. 9A). The results of the seven-step cancer-immunity cycle analysis showed that the high-risk patients had higher activity in step 1 (release of cancer cell antigens), while they had lower activity in step 4 (trafficking of immune cells to tumors) and step 5 (infiltration of immune cells to tumors) (Fig. 9B). These results revealed that there may be an immune-inhibited TME in the high-risk group. Then, the different analyses of IPS revealed that the low-risk subgroup had a higher IPS score than the high-risk subgroup, which implied that the low-risk subgroup might have a better response rate to immunotherapy than the high-risk subgroup (all P < 0.05; Fig. 9C). Additionally, we used the "IMvigor 210" dataset to verify this claim, and the results showed that the high-risk subgroup had a poorer OS than the low-risk subgroup (P < 0.001; Fig. 9D) and that the low-risk subgroup had a better response rate to atezolizumab than the high-risk subgroup (P = 0.03; Fig. 9E).

### The novel signature can predict drug sensitivity in bladder cancer

**Figure 10:**
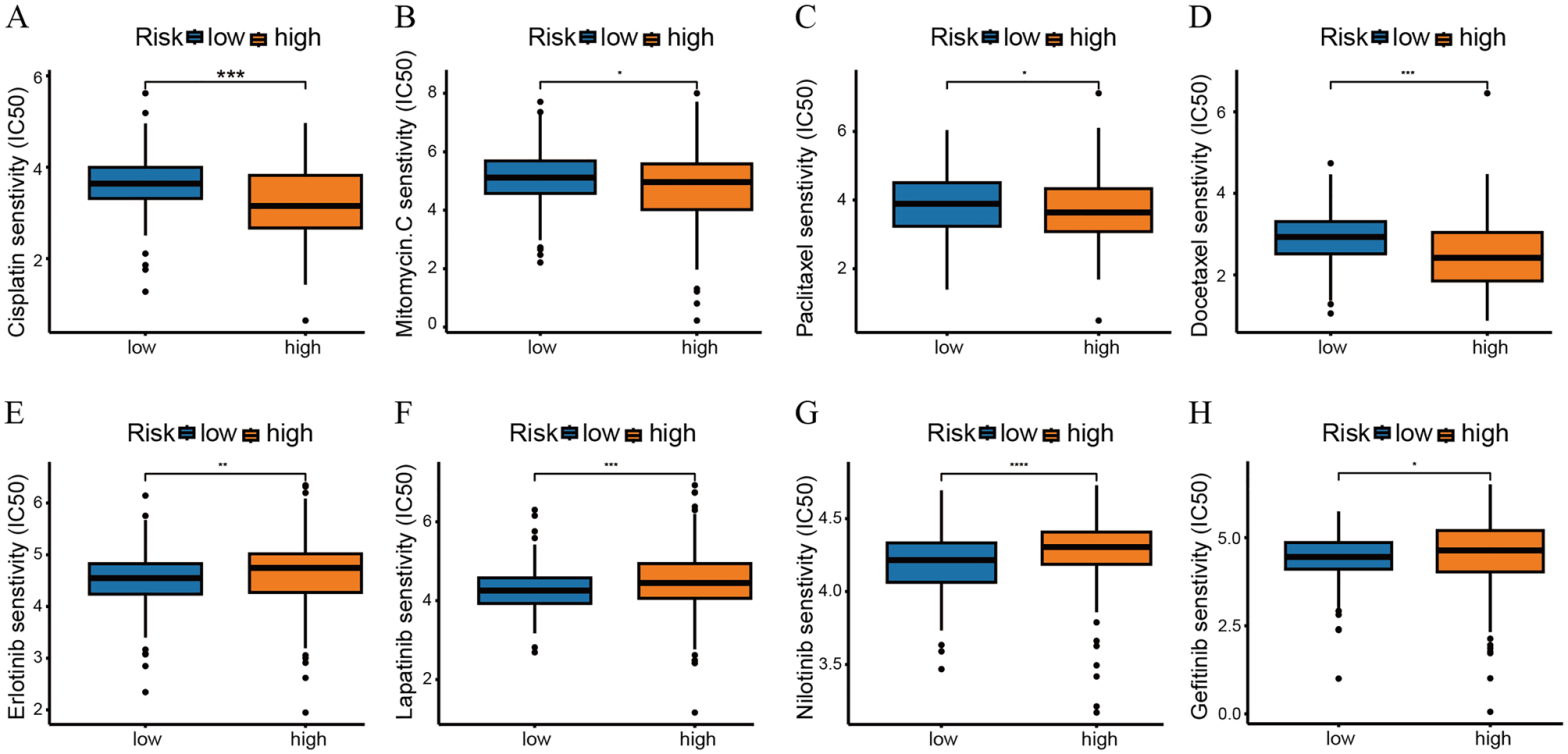
The novel signature can predict drug sensitivity. The different IC50 values of (**A**) cisplatin, (**B**) mitomycin-c, (**C**) paclitaxel, (**D**) docetaxel, (**E**) erlotinib, (**F**) lapatinib, (**G**) nilotinib, and (**H**) gefitinib.

We conducted a drug sensitivity analysis of commonly used chemotherapeutics and targeted drugs for BLCA and found that the high-risk group had a lower IC50 among common chemotherapeutics (including cisplatin (Fig. 10A), mitomycin-c (Fig. 10B), paclitaxel (Fig. 10C), and docetaxel (Fig. 10D)). However, it has a higher IC50 in common targeted drugs (including erlotinib (Fig. 10E), lapatinib (Fig. 10F), nilotinib (Fig. 10G), and gefitinib (Fig. 10H)). These results suggested that high-risk patients may benefit more from chemotherapy and that low-risk patients may benefit more from targeted therapy.

### Construction and evaluation of a prognostic nomogram

**Figure 11:**
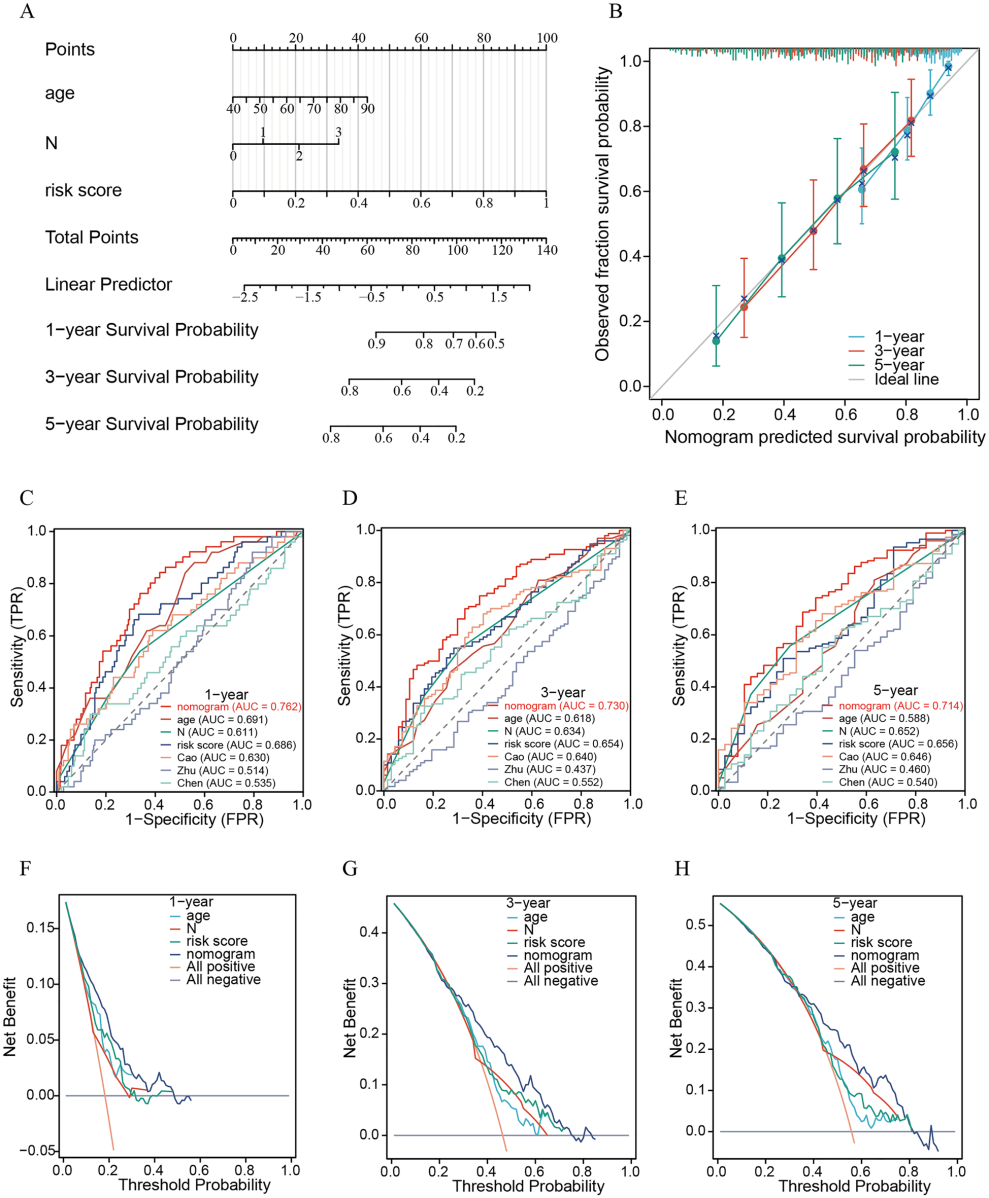
The nomogram was more precise in predicting the prognosis of BLCA patients than other signatures. (**A**) The prognostic nomogram constructed based on the risk score and clinicopathological parameters predicted the survival rate of TCGA-BLCA patients at 1, 3, and 5 years. (**B**) Calibration curves showing the concordance between predicted and observed 1-, 3-, and 5-year survival rates. AUCs of the nomogram, risk score and other signatures in ROC analysis were calculated at (**C**) 1-, (**D**) 3-, and (**E**) 5-year OS times in the TCGA-BLCA cohort. Decision curve analyses (DCA) for the nomogram, EETS and other signatures at (**F**) 1, (**G**) 3, and (**H**) 5 years to assess clinical utility in the TCGA-BLCA cohort.

Supplementary Fig. 4A displays the results of univariate analysis. Age, N stage, and risk score were defined as independent prognostic factors in the TCGA dataset using multivariate Cox regression (all P < 0.05; Supplementary Fig. 4B). Then, we combined age, N stage and risk score to construct a prognostic nomogram (Fig. 11A). Calibration curves demonstrated an excellent fit regarding the predicted versus observed 1-, 3-, and 5-year OS of the BLCA patients (Fig. 11B). Moreover, the ROC curves exhibited better predictive capability in the current nomogram to assess the 1-, 3- and 5-year OS than risk score, age, N stage and risk scores published by Zhu et al., Chen et al., and Cao et al. (the values of the nomogram’s AUCs at 1 year, 3 years, and 5 years were 0.762, 0.730, and 0.714, respectively; Fig. 11C–E). Additionally, DCA revealed the superiority of the current nomogram over risk score, age, and N stage in predicting 1-, 3-, and 5-year OS (Fig. 11F–H).

## Discussion

In this study, the prognostic role of *ENO1* in BLCA and its association with CD8+ T-cell exhaustion were analyzed by combining bulk RNA-seq and scRNA-seq data. In addition, we found heterogeneity among ECs with different *ENO1* expression levels and constructed a prognostic signature based on differentially expressed genes among ECs to predict prognosis and treatment responses.

First, through the TCGA-BLCA and GSE13507 data, we found that *ENO1* expression was upregulated in tumor tissue and positively associated with poor prognosis. The FPH cohort also confirmed this conclusion. In recent years, *ENO1* has been characterized by (1) cell surface localization; (2) high expression in cancer cells; and (3) its expression being positively associated with poor prognosis^18,45^, which has been considered a novel tumor marker and therapeutic target, consistent with our conclusion. In addition, some studies have shown that silencing *ENO1* expression can inhibit cancer invasion^46^, migration and metastasis^47^, which was also confirmed in vitro in our study.

Recent studies have found that T-cell dysfunction is associated with glucose metabolism disorders^48^, and the competition between tumor cells and CD8+ T cells for limited glucose leads to reduced CD8+ T-cell effector function^49^. However, Chang et al. found that in exhausted CD8+ T cells in vitro, it was difficult to increase their effector capacity even if superphysiological levels of glucose were given^50^. Interestingly, by single-cell sequencing data analysis of CD8+ T cells, we found that *ENO1* and inhibitory receptors such as *LAG3* were upregulated with the progression of CD8+ T-cell exhaustion in our study, which was consistent with the results of L.F. Gemta’s study and suggested that *ENO1* may undergo posttranslational modification in exhausted CD8+ T cells^17,51^.

*ENO1* can, through the regulation of the cell cycle^52^ and apoptosis, maintain cancer cell proliferation and resistance to cell death^53^, and through the induction of EMT, promote invasion and metastasis^54^. In the current study, we found significant heterogeneity between epithelial cells with different *ENO1* expression levels. ENO1^high^-BLCA cells promote BLCA progression mainly by affecting metabolism, the cell cycle and EMT. Then, the hub genes were defined as the intersection of differentially expressed genes between different ECs. Functional enrichment analysis showed that the hub genes were enriched in energy metabolism, apoptosis regulation, and multiple oncogenic pathways.

LASSO can effectively screen unimportant variables and prevent overfitting. Therefore, we chose LASSO to construct a prognostic signature. We used TCGA as the training set and GSE13507 as the validation set to construct a novel signature consisting of 6 genes by LASSO regression analysis. Then, BLCA patients were divided into high-risk group and low-risk group according to the risk score of each sample. The results showed that the risk score could successfully predict the OS of BLCA patients, and the OS of high-risk group was significantly worse than that of low-risk group. The ROC curve also indicated that the novel signatures had good prognostic ability. These results suggested that the *ENO1*-associated epithelial prognostic signature played a carcinogenic role in BLCA progression, which was consistent with the function of ENO1.

Considering the high tumor mutation burden of BLCA, immune checkpoint inhibitors (ICIs) have been an alternative treatment with promising results. However, the clinical efficacy of ICI therapy for BLCA patients is far from satisfactory, with a response rate to ICIs as low as 20–30%^55–57^. The lack of precise biomarkers to predict the response to ICIs might be quite a reason; therefore, it is urgently needed to identify reliable prognostic biomarkers to increase the proportion of responders to ICIs in BLCA. In this study, the novel signature was also found to be correlated with IPS. This result indicated that the current signature might be taken as an alternative signature to predict the response to ICIs, which was confirmed by the IMvgior 210 cohort, which consisted of 298 urothelial carcinoma patients receiving atezolizumab (a PD-L1 inhibitor). However, these findings need further validation.

There were several shortcomings in our study. First, considering that consensus was not reached on CD8+ T-cell exhaustion genes, we selected exhaustion genes from the published records, which would result in selection bias and publication bias. Second, the novel signature exhibited good prediction of prognosis and treatment response, but it would be better if it was verified by the cohort from our center. Third, single-cell analysis revealed the relationship between *ENO1* and CD8+ T-cell exhaustion using a public dataset, and we will continue to work on these in vitro and in vivo studies.

## Conclusion

In conclusion, by integrating the results of bulk RNA-seq, protein, and scRNA-seq, our work revealed that *ENO1* was a prognostic marker and might participate in CD8+ T-cell exhaustion within BLCA. Then, there was heterogeneity between ECs with different *ENO1* expression levels, and based on these differentially expressed genes, we constructed a novel signature with good prediction of prognosis and treatment response among BLCA patients.

### Supplementary Information

Supplementary Table 1.

Supplementary Table 2.

Supplementary Table 3.

Supplementary Table 4.

Supplementary Information 5.

## Data Availability

Publicly available datasets were analyzed in this study. These data can be found here: TCGA (http://portal.gdc.cancer.gov/) and GEO (www.ncbi.nlm.nih.gov/) under the accession numbers GSE13507, GSE135337 and GSE149652. The RNA-seq profiles and clinical data of the “IMvigor 210” cohort were derived from http://research-pub.gene.com/IMvigor210CoreBiologies/.
