# Supplementary material for: Multi-omics reveals the role of ENO1 in bladder cancer and constructs an epithelial-related prognostic model to predict prognosis and efficacy
Source: Sci Rep. 2024 Jan 25;14:2189. doi: 10.1038/s41598-024-52573-8 (PMC10811216; doi:10.1038/s41598-024-52573-8)
Supplement: Supplementary file 5 — Supplementary Information 5. [file 41598_2024_52573_MOESM5_ESM.docx]

Supplementary Material

# 1 Supplementary Tables

**Supplementary table 1.** Clinical characteristics of the data sets used in this study.

**Supplementary table 2.** The siRNA sequences targeting ENO1 and the specific primer sequences of ENO1.

**Supplementary table 3.** The hub genes.

**Supplementary table 4.** The results of univariate Cox regression of the hub genes.

## 2 Supplementary Figures

**
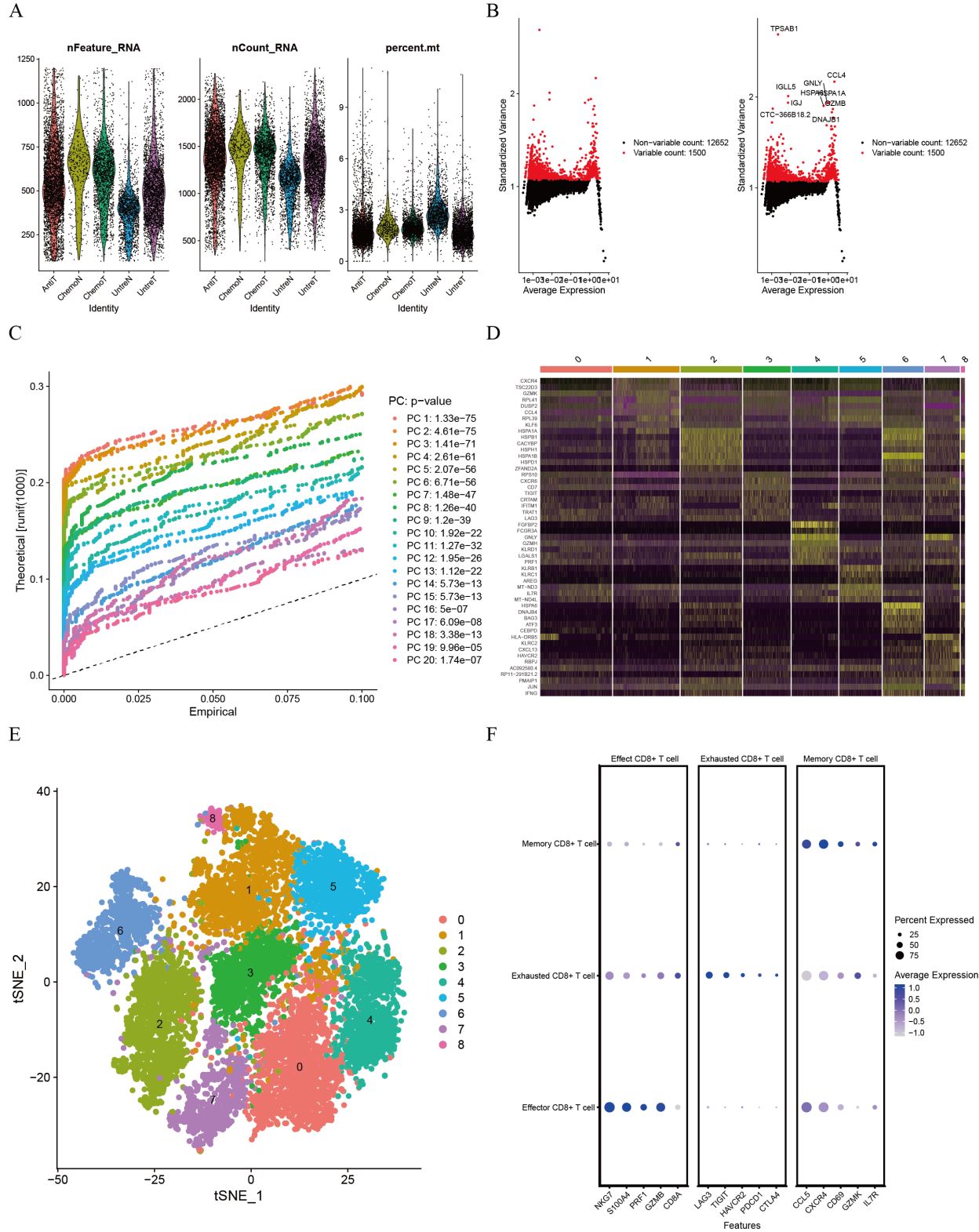
**

**Supplementary Figure 1.** Quality control process of single cell analysis in GSE149652. **(A)** Quality control plots of cell samples. **(B)** 1500 variable genes and the top 10 variable genes across cell samples were identified. **(C)** PCA was conducted to reduce the dimension of data sets. **(D)** Heatmap illustrated the distribution of different clusters marker genes. **(E)** [CD8+ T](javascript:;) cells were classified into 9 clusters with the t-SNE algorithm in GSE149652. **(F)** The bubble plot exhibited the expression of the markers of cell subtypes.


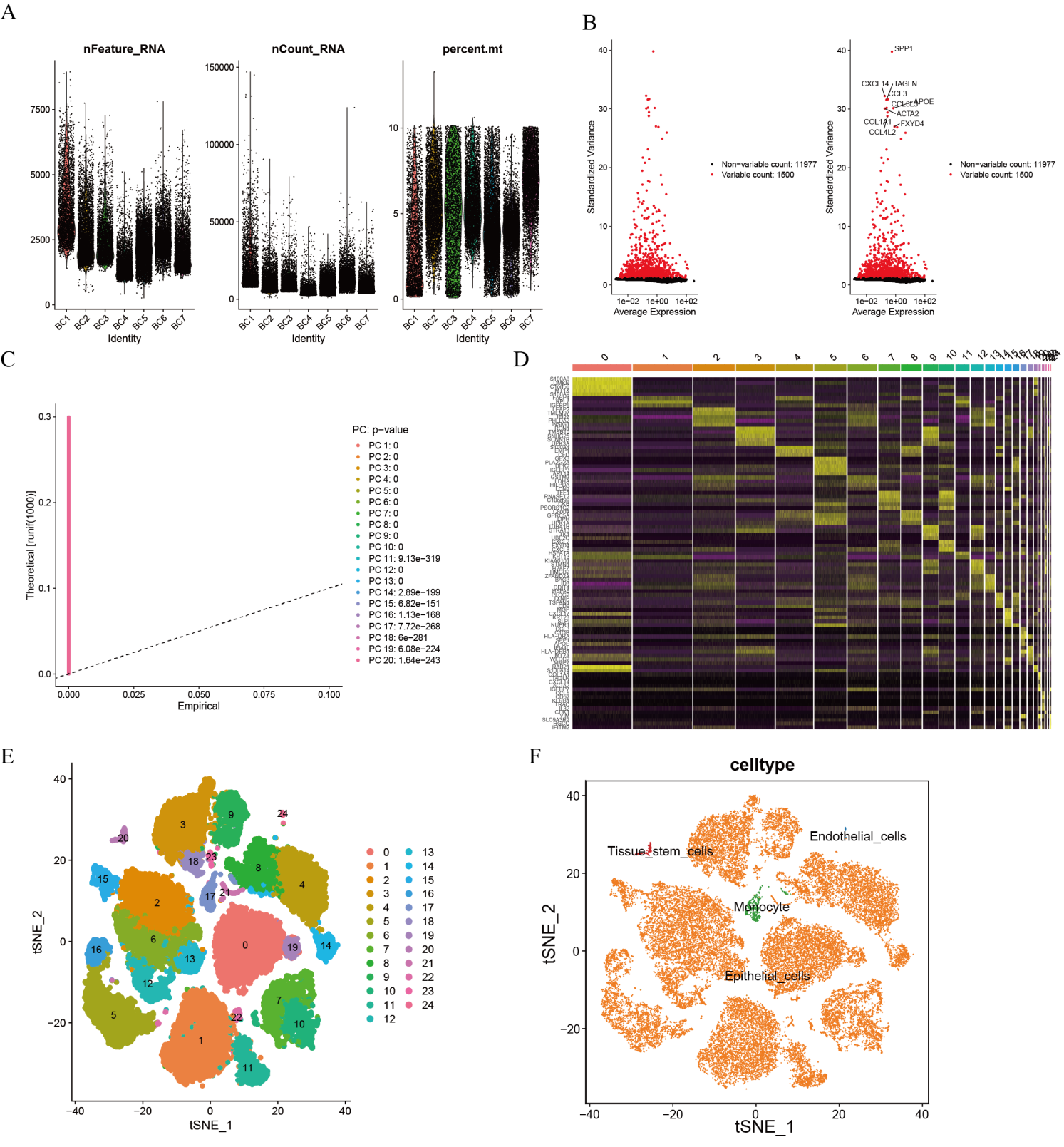


**Supplementary Figure 2.** Quality control process of single cell analysis in GSE135337. **(A)** Quality control plots of cell samples. **(B)** 1500 variable genes and the top 10 variable genes across cell samples were identified. **(C)** PCA was conducted to reduce the dimension of data sets. **(D)** Heatmap illustrated the distribution of different clusters marker genes. **(E)** All cells were classified into 25 clusters with the t-SNE algorithm in GSE149652. **(F)** Annotations for cell subtypes in GSE149652.

**
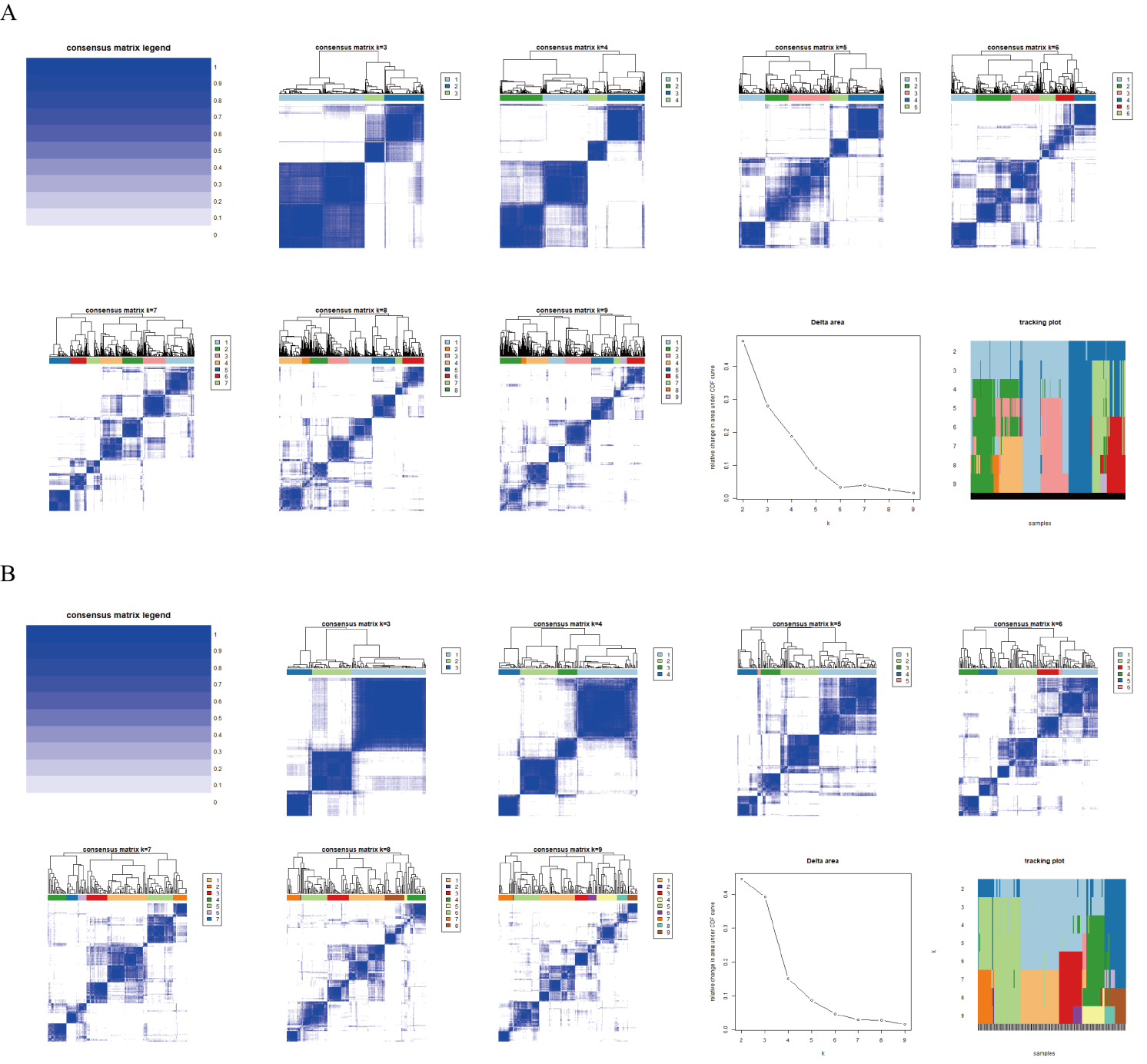
Supplementary figure 3.** The results of Consensus clustering analysis. **(A)** The consensus heatmap, relative change in area under CDF curve and tracking plot in TCGA cohort. **(B)** The consensus heatmap, relative change in area under CDF curve and tracking plot in TCGA cohort.


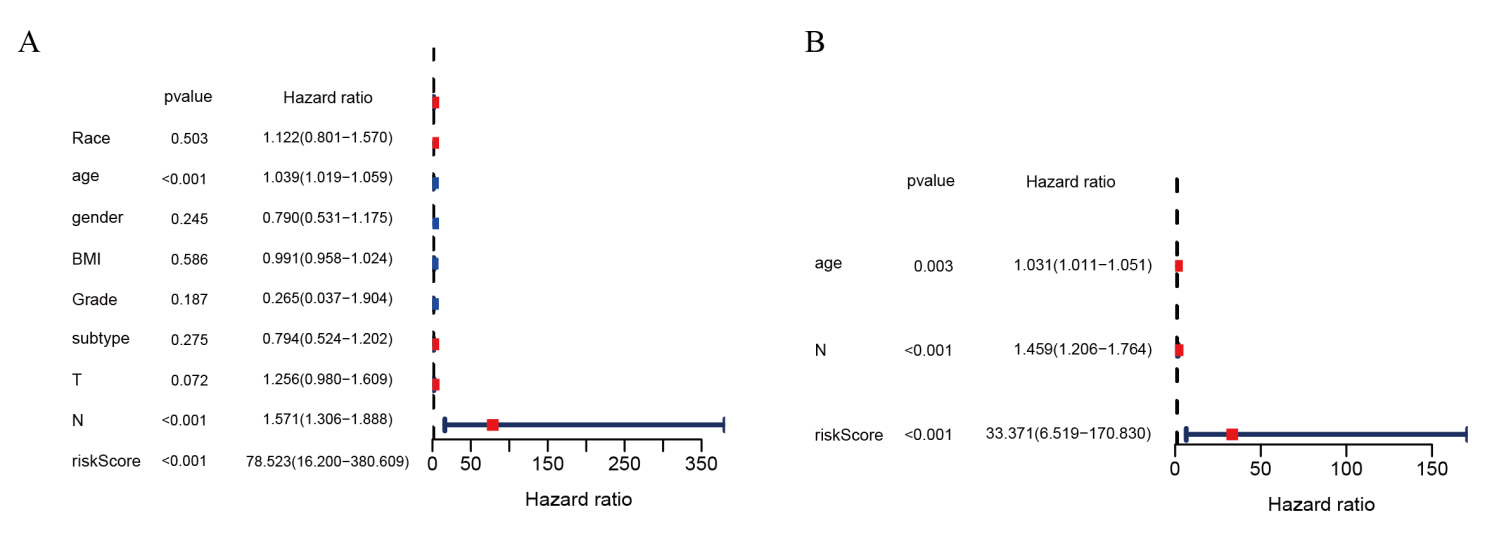


**Supplementary figure 4.** Univariate and multivariate Cox regression. **(A)** Forrest plot of the univariate Cox regression analysis in TCGA cohort. **(B)** Forrest plot of the multivariate Cox regression analysis in TCGA cohort.


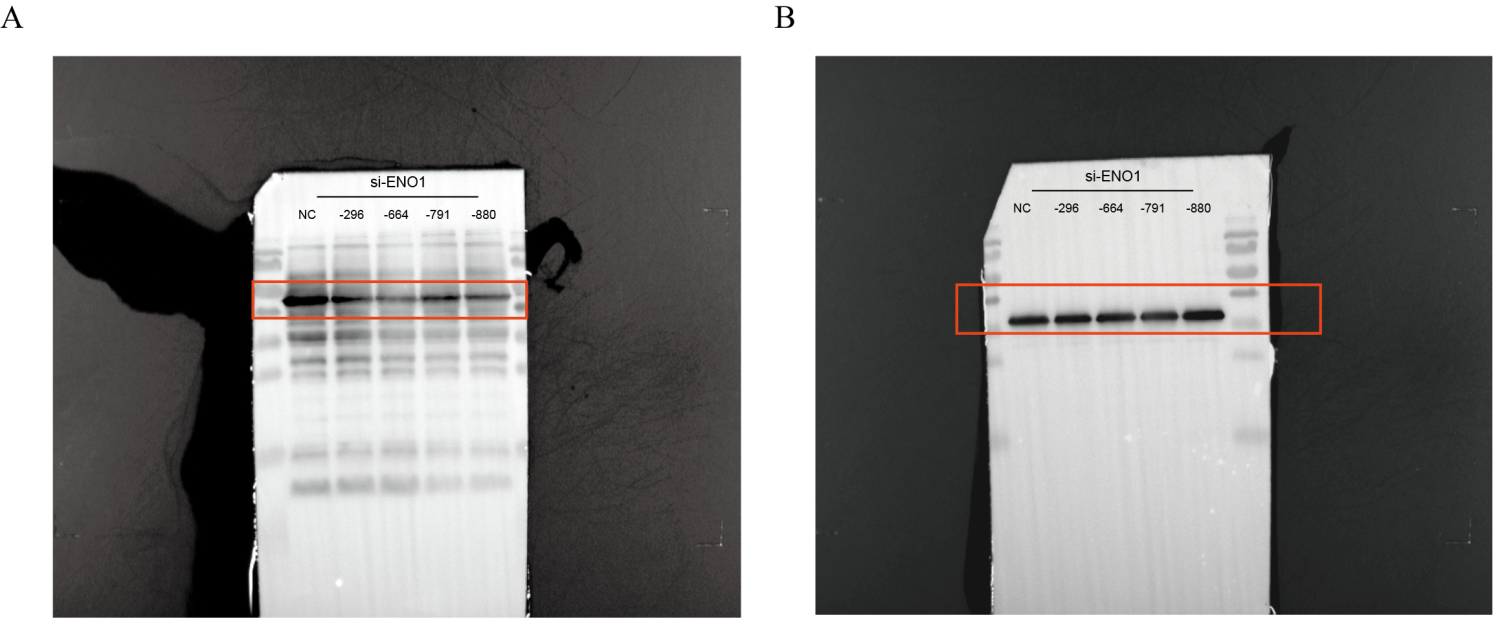


**Supplementary figure 5.** The original images of western blots for figure 5A. (A) The original western blot image of ENO1. (B) The original western blot image of GAPDH.
